# Enhancing Orthodontic Renewal and Retention Techniques: A Systematic Review

**DOI:** 10.7759/cureus.58843

**Published:** 2024-04-23

**Authors:** Nawaf H Al Shammary

**Affiliations:** 1 Department of Preventive Dentistry, College of Dentistry, University of Ha'il, Ha'il, SAU

**Keywords:** ohip-14, heath related quality of life, dental malocclusion, retainer, renewal - retention redundancy - orthodontics - systematic review - saudi arabia

## Abstract

Orthodontists have a variety of options available for retainers. Research in Orthodontics focuses on assessing outcomes important to clinicians; however, there is inconsistency in how these outcomes are selected and evaluated. This review sought to assess the effects of different orthodontic retainers on patients’ quality of life (QoL). Various approaches were employed in this systematic review, and a thorough search was conducted across six databases. The review involved a comprehensive evaluation of six included studies, highlighting changes in dental structure post-treatment, emphasizing the role of extraction procedures and the quality of debonding in improving retention. The study identified key outcomes for orthodontic clinical trials, highlighting orthodontists' preferences for specific retainer types. Moreover, it discussed the impact of sociocultural influences on retention care. Involving patients actively in discussions about whether to end or extend the retention phase was deemed essential. Noteworthy improvements in occlusal outcomes were linked to extraction treatments. Gender and malocclusion severity influenced QoL before and after orthodontic treatment. The degree of improvement observed in the Class III malocclusion group was comparatively lower than that in the Class I and Class II groups. Orthodontic treatment was found to yield favorable psychological outcomes, as evidenced by notable enhancements in self-esteem and social engagement among individuals. Fixed appliances were shown to negatively affect oral health-related quality of life (OHRQoL), particularly for those with aesthetic and functional concerns. A consensus has been reached on the essential themes and outcomes that should be incorporated in clinical trials related to orthodontic retention for non-cleft and non-surgical cases.

## Introduction and background

Orthodontic treatment typically concludes with a phase known as retention. Relapse refers to the reappearance of specific malocclusion issues that were previously corrected through orthodontic intervention [1]. The term 'relapse' is also used to describe the occlusal alterations due to aging, even in individuals who have not undergone orthodontic treatment [2-3]. There is evidence to suggest that patients who have had orthodontic treatment often experience some degree of relapse [4]. To prevent this, orthodontic retainers are utilized to maintain the corrected alignment of teeth following the treatment phase. It is common for many orthodontic patients to be advised to wear retainers after the removal of their orthodontic appliances [5,6].

Orthodontic practitioners have a variety of retainer options to select from: detachable retainers (removable) and fixed retainers [7,8]. The two most frequently utilized types of removable retainers (RRs) are the Hawley retainer and the vacuum-formed retainer (VFR). On the other hand, fixed retainers, also known as bonded retainers (BR), are frequently recognized in the field of dentistry. Each type of retainer has its own distinct advantages and limitations. Several randomized clinical trials (RCTs) have been undertaken to investigate the effectiveness of different retainers and retention techniques [9-11].

Despite limited data availability supporting retention practices, a thorough examination of existing literature has yielded significant insights for healthcare practitioners and patients seeking orthodontic treatment [12]. Over the past few decades, there has been a focused effort to enhance the empirical foundation for healthcare decisions, leading to an increase in the number of RCTs and systematic reviews (SRs) [13-15].

The field of orthodontics has notably embraced these widely recognized research methodologies, sometimes referred to as the 'gold standard.' Incorporating data derived from RCTs has become increasingly common in the field of orthodontics [16] to enhance the decision-making processes. A significant component of these trials focuses on comparing the effectiveness of different treatments. SRs and Cochrane reviews in orthodontic treatment heavily rely on data extracted from RCTs to conduct their meta-analyses. Therefore, the meticulous selection, precise measurement, and detailed reporting of outcomes play a crucial role in trial methodology [17-19]. It is essential that any substantial alterations in study outcomes precisely represent the impact of the varying treatments. To conduct a comprehensive evaluation of the most advantageous interventions, it is imperative to take into account the perspectives of healthcare providers, patients, and funders [20]. Consistent outcome measures are crucial, especially in meta-analyses, as they provide precise estimations of effects and aid in the development of reliable conclusions. Consequently, this process enables individuals to make informed decisions based on the most credible and trustworthy information available [1,3,8-11].

In spite of recent developments in research methodology, there are significant limitations in the field of orthodontic research [18]. Evaluating interventions presents several challenges, such as inconsistency in outcomes [19], the presence of bias in reporting outcomes in clinical trials and SRs, and the emphasis on measuring morphological effects of treatment that may be more important for providers than for patients [19]. The concept of quality of life (QoL) refers to an individual's personal assessment of their well-being, influenced by their satisfaction or dissatisfaction with different aspects of life that are important to them. Currently, there is a growing interest in exploring the association between malocclusion, the need for orthodontic treatment, and health-related QoL [7]. Traditionally, the assessment of orthodontic treatment need and its outcomes has relied on malocclusion models and cephalometric radiographs [20-22].

The assessments were formulated from the orthodontist's perspective, often overlooking the patient's perspective. However, it is imperative to consider the viewpoint of the patient, as it may differ from that of a healthcare practitioner, offering unique perspectives that complement conventional clinical evaluations [11]. Recently, the WHO has suggested incorporating patient-centered assessments of QoL into clinical research [22-26]. A comprehensive assessment of malocclusion involves exploring its psychological, social, and physical impacts, as well as its influence on an individual's QoL, providing a more precise view of their overall well-being [21-28]. There is a notable lack of scholarly literature on assessing the QoL in patients needing orthodontic treatment for malocclusion [29]. Therefore, the primary aim of this systematic review was to assess and present key outcomes such as patients’ QoL with various types of orthodontic retention treatments. The aim was to comprehensively analyze existing research on the topic, pinpointing any gaps or unexplored areas within the existing literature to enhance understanding and practice.

## Review

Methods

The primary aim of this SR was to evaluate and present the main outcomes, such as the QoL of patients, resulting from various types of retention applied by orthodontists. All studies included in this review were carefully selected in accordance with the guidelines of the Preferred Reporting Items for Systematic Reviews and Meta-Analyses (PRISMA). These guidelines, established and refined earlier for structuring SRs [30], involve formulating a specific question using the PICO model: Participants (individuals undergoing orthodontic retention treatment), Intervention (implementation of quality care and retention programs), Control (evaluation of quality care and retention programs with positive outcomes), and Outcome (attainment of successful and effective retention treatment).

Search strategy

The SR focused on specific terms such as "retention," "retainer," "orthodontics," "occlusal," and "orthodontists," all classified under the Medical Subject Headings (MeSH). Additional synonyms were also employed in the search. When utilizing the Web of Science (WOS), the search terms included "retention" and "retainer" in relation to "orthodontics," and "occlusal" in relation to "orthodontists." The selected articles were published between 2018 and 2023. Table 1 presents the search strategy in the databases used.

**Table 1:** Search strategy in databases. MeSH: Medical subject headings.

Database	Search terms
National Library of Medicine (NLM), the Cochrane Central Register of Controlled Trials (CENTRAL), the PubMed Central (PMC).	“retention”[MeSH Terms], “retainer”[MeSH Terms], “orthodontics”[MeSH Terms], “occlusal”[MeSH Terms], “orthodontists”[MeSH Terms]
WOS	retention and retainer on orthodontics for occlusal by orthodontists

In order to meet the inclusion criteria, articles must be originally written in English, without translations, and should have been published within the past five years. They should focus on quality-of-care and retention programs for orthodontists and be relevant to the field.

Conversely, studies conducted in contexts other than quality of care and retention programs were excluded. Additionally, any studies focusing on the prenatal period were not considered. Furthermore, review articles, SRs, scoping, or narrative reviews were excluded from this search.

The primary data sources utilized were Medline, CINAHL, PsychInfo, Saudi Digital Library, Science Direct, Cochrane Central Register of Controlled Trials (CENTRAL), and PubMed. By employing Boolean operators (AND, OR, NOT), the search identified 6,109 references. Specifically, 2,145 were from Medline, 1,906 from CINAHL, 1,059 from PubMed, 612 from CENTRAL, 114 from PsychInfo, and 273 from Science Direct. These data contained a wealth of valuable information.

Efforts were made to ensure that the retrieved literature was accurate and current by applying date filters, focusing on the last five years across all databases. The search was performed from August 2023 to December 2023. Papers written in languages other than English, as well as those not available in full text, debates, conference abstracts, and dissertations were excluded from the study. Nevertheless, reference lists were thoroughly checked to identify relevant studies. After screening the results and removing non-English papers, duplicates, SRs, and meta-analyses, it was evident that 83% (5070) of these retrieved studies had been overlooked, with an additional 16.4% (983) excluded based on filtering. Ultimately, only one hundred and one papers were deemed suitable for further analysis.

Data extraction and analysis 

The reviewers effectively extracted and analyzed data from the full texts of the selected articles, covering various aspects such as general information, introduction, study location, criteria for selecting the quality management system, and authorities used in the study, analytical methods, type of guidelines, discussion of data conclusions, future perspectives, and limitations of the studies. Clarification through discussions between the two reviewers resolved any confusion regarding the eligibility of the studies, ensuring the most reliable and eligible results for subsequent discussion [31].

Assessment of risk of bias (RoB)

The Agency for Healthcare Research and Quality (AHRQ) checklist for evaluating RoB in Comparative Effectiveness Reviews was utilized [32]. This process was performed to consider assumptions and limitations when assessing validity and generalizability.

Results and discussion

**Figure 1:**
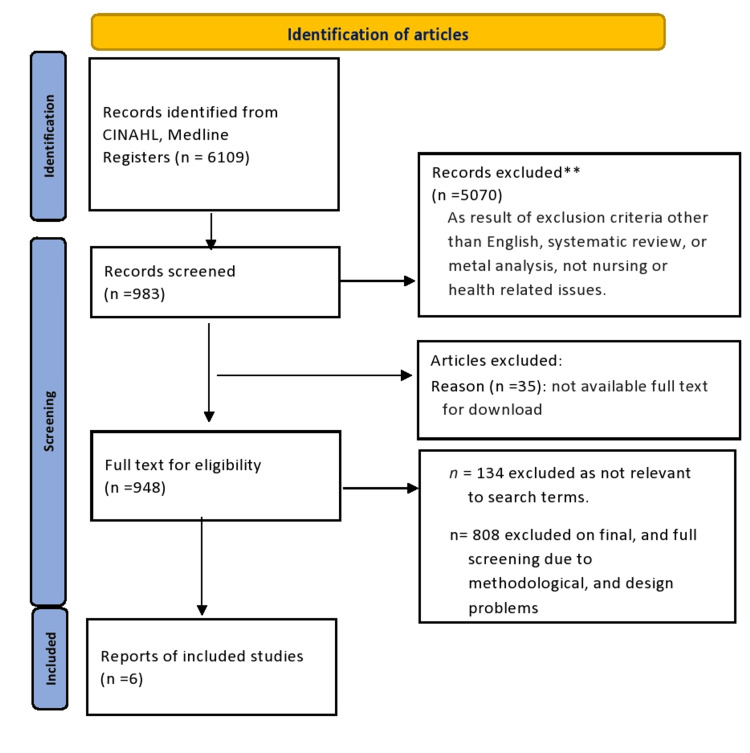
The PRISMA flowchart. PRISMA: Preferred Reporting Items for Systematic Reviews and Meta-Analyses.

About 5,070 articles were excluded due to the unavailability of the full text. The remaining articles underwent a review for relevance, with 983 articles then scrutinized for quality following initial screening, and subsequently re-evaluated for relevance. Out of these, 134 articles were excluded for not meeting the search criteria, and 808 were excluded during the final screening phase due to methodological and design issues. Ultimately, only six articles remained for review, selected based on their titles closely aligning with the original search terms (Figure 1).

Table 2 summarizes the six study matrices to illustrate the main findings and aims of the included studies in this review.

**Table 2:** The six included studies illustrate the main findings and aims in this review.

Author, year	Title	Aim of the study	Country	Study design
Carneiro et al. (2022) [33]	Retention decisions and protocols among orthodontists practicing in Canada: A cross-sectional survey.	to investigate retention techniques and retainer characteristics utilized by orthodontists	Canada	A cross sectional study using online questionnaire for 576 orthodontists.
Lasance et al. (2020) [34]	Post-orthodontic retention: how much do people deciding on a future orthodontic treatment know? and what do they expect? A questionnaire-based survey	-To examine the extent of information and expectations held by individuals who are making decisions regarding future orthodontic treatment in relation to post-orthodontic retention. -To analyze the potential impact of sociocultural variables on these knowledge and expectation levels.	Germany	A cross-sectional study used questionnaire administered to a sequential sample of 220 individuals who served as legal decision makers prior to their initial assignment.
Tsichlaki et al. (2020) [35]	Development of a core outcome set for use in routine orthodontic clinical trials	To provide a fundamental set of outcomes that can be utilized in clinical studies focusing on orthodontic therapy, excluding patients with cleft or orthognathic conditions.	United Kingdom	A comprehensive examination of the existing orthodontic research literature was conducted Qualitative interviews and focus groups were employed to gather supplementary outcomes of significance to patients, specifically targeting adolescents within the age range of 10 to 16 years.
Angst et al. (2021) [36]	Stability of occlusal outcome during long-term retention: the time-dependent variation of the ABO index	To identify any potential risk factors associated with these occlusal alterations.	United States	A retrospective cohort study to evaluate the occlusal changes that occur at the time of debonding and a median of 8 years thereafter (during the retention period) using the ABO objective grading system.
Grewal et al. (2019) [37]	Psychological impact of orthodontic treatment on quality of life–a longitudinal study	To evaluate the psychosocial, functional, and cosmetic transformations experienced by young people before and after orthodontic treatment. To evaluate the impact of orthodontic appliance therapy on the quality of life of both male and female individuals with malocclusion.	India	A longitudinal study consisted of a sample of young adults aged 18.1 to 25.3 years, which was selected using a convenience sampling method. The total of 400 participants involved. The study examined the impact of dental aesthetics on self-perception before and after treatment, considering gender and the severity of malocclusion (Angle's class I, II, III). The Psychosocial Impact of Dental Aesthetics Questionnaire (PIDAQ) was utilized, with modifications made to suit the specific needs of the Indian ethnic group.
Paes da Silva et al. (2020) [38]	Oral health-related quality of life in orthodontics: a cross-sectional multicentre study on patients in orthodontic treatment	To evaluate the impact of several factors on the OHRQoL among orthodontic patients across different age groups, including children, adolescents, and adults.	Germany	A cross-sectional study was conducted using an online questionnaire, which included the German version of the OHIP-G14 as a tool to evaluate OHRQoL. Participants were also required to complete an additional set of 23 items.

Upon reporting the main findings of these selected studies for relevance to this SR, the six studies could be categorized into three main themes: 'Retention Decisions by Orthodontists,' 'Valuable Outcomes from Routine Trials,' and 'Quality-of-Life (QoL) Measurements.' The following sections summarize and discuss the main findings of the included studies.

Retention Decisions by Orthodontists

Carneiro NC et al. (2022) undertook a study in which 101 questionnaires were collected and completed, yielding a response rate of 18.0%. The majority of respondents were male orthodontists, accounting for 74.3% of the total. A considerable percentage of individuals (62.4%) obtained their specialized certification in Canada, while 35.6% completed their training in the United States. Nearly all participants were working (96.0%) in private practice. The mean duration of experience as an orthodontist was found to be 19.7 years, with a standard deviation of 12.0 years. The VFR orthodontic retainer was the preferred choice for the maxilla, exhibiting a usage rate of 50.5% among orthodontists. In contrast, the BR retainer was more commonly used for the mandible, accounting for 54.5% of cases. It was noted that orthodontists often combine a BR and a VFR in the maxillary arch for orthodontic re-treatments. This approach aids in maintaining the alignment of previously impacted anterior teeth and closing any existing interdental spaces.

The findings of this study indicate that a significant proportion of orthodontists, with 66.3% in the maxilla and 63.0% in the mandible, are in favor of implementing a full-time wearing schedule for orthodontic appliances. The probability of orthodontists supporting the initial full-time wearing schedule significantly increased when using a rapid palatal expander (RPE) along with braces (83.6% vs. 16.4%; P<0.001). In contrast, there was a decreased tendency among orthodontists to recommend the initial full-time usage schedule when the RR was accompanied by a BR in the lower jaw (71.4% vs. 28.6%; P<0.074).

Regarding the upper jaw, a common wear practice adopted by orthodontists is to wear the appliance indefinitely at night and/or during sleep, with a prevalence rate of 40.6%. Similarly, in the lower jaw, the most commonly selected approach was to wear the appliance indefinitely during the night or while sleeping, with a prevalence rate of 36.6%.

This study represents the first investigation into orthodontic retention procedures within the Canadian context. The careful selection of a retainer and the implementation of its usage are important factors to be considered both before and after orthodontic treatment.

An online survey was conducted to explore how orthodontists in Canada retain teeth using fixed and detachable retainers. The survey found that 14.5% of registered orthodontists provided responses. Employing a pre-validated survey questionnaire allows for comparing responses from studies conducted across several countries. The results of this study indicate significant variation in how orthodontists select retainers and methods to maintain the position of teeth post-orthodontic treatment.

The VFR has become a popular choice for the upper teeth, mainly due to its manufacturing simplicity, which greatly influences its widespread preference. Many orthodontists reported that they manufacture their VFRs in-house at their dental practices.

A significant proportion of individuals selected the pressure-forming technique instead of the more common vacuum-forming method used in Australia. The most popular prefabrication thickness for VFR chosen by individuals was 1 mm, consistent with findings from similar studies in various countries. However, variations in the materials used for VFR among orthodontists indicate the need for further studies to determine the best material type, thickness, and production technique. For mandibular retention, the buccal retractor (BR) emerges as the best choice among orthodontists.

Identifying individuals with a proclivity for mandibular incisor overlap, commonly seen in age-related occlusal changes, can influence the choice of retainer used for treatment. The potential advantages of aesthetics and the recognition that patients may not always comply with wearing RRs long-term suggest that BRs might be a better option. The main bone remodeling patterns in the upper jaw are similar to those found in Australia and New Zealand. Specifically, the attachment of the buccal tube to just the four incisors is typical. However, this differs from the method utilized in the Netherlands, where the BR includes not only the four incisors but also the canines. Orthodontists modify their preferred choice of retainer based on specific clinical needs. A study found that a combined RR and BR retainer, known as a hybrid or dual retainer, was preferred for re-treatment in the maxilla and affected anterior maxillary teeth. This suggests that orthodontists are concerned about the risk of relapse based on specific malocclusion traits. It is crucial to emphasize that this implies the adoption of various retention strategies, recognizing the limitation of a standardized retention approach. Orthodontists typically evaluate retention for 1-2 years after removing orthodontic equipment. Many orthodontists recommend indefinite retainer wear for patients. However, some orthodontists expressed concerns about general dentists conducting retainer check-ups. The mean period of professional experience among orthodontists was 19.7 years, with those who have more experience being less likely to support indefinite retainer wear. When selecting a retainer, orthodontists primarily consider the condition of the teeth before treatment. This finding is consistent with previous research conducted in various countries. The recent guidelines offer significant insights for both practitioners and patients. However, it is crucial that the selection of retainers and the implementation of retention measures are grounded in evidence-based dentistry principles. Therefore, the incorporation of clinical research, patient viewpoints, and practitioner expertise is necessary for a comprehensive approach. One limitation identified in the survey is the significantly low response rate of participants. Implementing additional recruitment strategies, such as the employment of paper-based surveys and making follow-up phone calls, could have potentially increased participation levels. The COVID-19 pandemic has presented challenges in engaging orthodontists in national and regional orthodontic conferences. However, the results of this study provide significant insights into the importance of orthodontic retainers in preserving tooth alignment within the Canadian context.

The website provides orthodontists with significant insights, allowing them to acquire knowledge of the orthodontic practices of their colleagues. Moreover, it has the potential to make a valuable contribution to advancing scholarly investigations and forming evidence-based recommendations for clinical care in the future [33] .

Lasance SJ et al. (2020) conducted a study where 220 surveys were completed, resulting in a participant return rate of 96.9% [34]. However, some respondents did not answer all questions, leading to variations in the number of responses across different topics. The number of responses varied from 198 to 218 out of 227 responses. At the individual question level, response rates ranged from 87.2% to 96.0%. The research involved 220 individuals, with an average age of 37.1 years. The researchers calculated a standard deviation of 11.9 years in ages, with participants ranging from 16.0 to 68.0 years old. The study found that a significant proportion of the participants were female (62.7%), Swiss nationals (74.8%), had a family history of orthodontic treatment (62.2%), and actively sought orthodontic advice (56.3%). Additionally, 46.3% of participants demonstrated knowledge about using retention appliances following orthodontic treatment. Only 32.6% of participants believed that retention was universally imperative, as opposed to being relevant solely in particular circumstances. The findings suggested that 52.8% of participants emphasized the importance of achieving a perfect orthodontic outcome for ensuring the enduring stability of the achieved results. Moreover, 77.8% of participants believed that teeth could shift independently of any orthodontic treatments. Regarding orthodontic retention expectations, data revealed that only 12.7% of participants preferred a retention period of less than one year, while 38.5% endorsed a 1 to 3-year retention period. Furthermore, 48.8% supported a retention period exceeding 3-10 years, or even a lifelong duration. The majority of participants (94.5%) considered orthodontic outcomes stability as highly important or important. Most participants (67.2%) favored BRs over detachable retention equipment, and 57.7% agreed that recalls should take place every 3 to 6 months. In contrast, a considerable proportion (31.0%) recommended a yearly recall frequency. Participants largely felt that orthodontists bore the greatest responsibility for ensuring post-orthodontic stability, followed by patients, and least by general dentists. The viewpoints mentioned were depicted by proportions of 73.2%, 50.0%, and 25.0% respectively. Ultimately, a substantial majority of the participants reached a consensus that it is reasonable to charge fees for follow-up visits necessary during the orthodontic retention phase, with 72.9% in agreement. The demographic and sociocultural characteristics of the participants greatly influenced the responses collected from the questionnaire.

Initial findings suggested that individuals with family members who had previous orthodontic experiences were more likely to understand the importance of using retention appliances after completing orthodontic treatment.

In contrast, those with a moderate or advanced level of education were less likely to believe that attaining a perfect orthodontic outcome can lead to long-term stability. Participants from Switzerland showed a higher tendency to agree to lifelong retention. Moreover, the study revealed that younger participants, individuals with a moderate or higher education levels, and those with family members who had undergone orthodontic treatment previously were more inclined to believe that teeth could undergo spontaneous movement without orthodontic appliances.

In conclusion, female participants and individuals with minimal education were more in favor of agreeing to charges related to retention recall visits.

This study represents the first effort to conduct a scientific investigation on individuals prior to their orthodontic consultation to assess their knowledge and expectations of post-orthodontic retention, as they navigate decisions about future orthodontic treatment.

Prioritizing individuals responsible for making decisions concerning future orthodontic treatment with prolonged retention is essential. This study aimed to determine participants' knowledge and expectations at the time they consented to the therapy.

Despite a sufficient number of participants and a satisfactory response rate, the study opted to utilize exclusively observational methods, refraining from hypothesis-driven methodologies, limiting the analysis to simple descriptive statistics.

One noteworthy finding was the identification of a minority (particularly, 46.3 percent) of prospective patients or their caregivers who were knowledgeable about the use of retention appliances following orthodontic treatment.

Given the widespread use of retention measures in orthodontic patients, and the significant emphasis on post-treatment stability (94.5%), this survey highlights a notable gap between participant expectations and observed clinical outcomes. Involving a family member who has previously undergone therapy seems to significantly enhance awareness about the importance of post-treatment maintenance.

However, a considerable proportion of participants still lack awareness regarding the importance of using retention devices. Another noteworthy observation is the discrepancy in participants' understanding of tooth movement and stability. While a substantial percentage (77.8%) demonstrated awareness of teeth moving without orthodontic treatment, many still held the belief that attaining an ideal outcome guarantees long-term stability. Only a small proportion (14.9%) acknowledged the importance of retention equipment in all situations.

The ongoing migration process could potentially affect the stability of previously attained orthodontic outcomes. This study shows that individuals with higher levels of education possess a similarly high level of knowledge. However, the concept of non-orthodontic tooth movement seems to be challenging for many individuals. Therefore, it is crucial to communicate this knowledge, preferably before the cessation of retention mechanisms.

A lack of understanding regarding tooth migration in adult orthodontic treatment has been observed. Only 13.2 percent of participants showed awareness of the importance of maintaining orthodontic outcomes. Analysis using logistic regression revealed that several sociocultural factors, such as age, family history of orthodontics, and level of education, significantly influence understanding of tooth movement and stability. However, these influences were mostly moderate, except for the impact of educational level. Recent surveys in Switzerland indicated that orthodontists generally choose to retain all patients subsequent to orthodontic treatment [39]. It was found that decision-makers in orthodontic treatment believe that only a small percentage of patients necessitate retention devices. This highlights the need for better patient education. Patient satisfaction with orthodontic treatment is strongly correlated with their expectation of stability. Understanding these expectations can help reduce dissatisfaction in future instances. It was found that most participants place considerable significance on attaining consistent outcomes but have diverse views on how long information should be retained, how often it should be reviewed, and how it should be accessed. A majority of the participants, specifically two-thirds of them, preferred BRs to removable ones. However, this study did not explore how sociocultural factors may influence this preference. Regarding retention duration preferences, only one-third of prospective orthodontic patients or their guardians believed in indefinite retention, while most anticipated a retention period of 1 to 10 years.

The current body of academic research lacks adequate empirical evidence to ascertain the best timeframe for retention. As a result, the decision on how long the retention period should last is mainly left to the expertise of the orthodontist. Upon closely examining existing scholarly works, a predominant trend towards the concept of lifetime retention becomes evident. However, this finding has substantial implications for patients regarding the frequency of follow-up appointments and the level of commitment required. The findings of the latest survey indicate that a significant number of individuals considering orthodontic treatment in the near future do not see lifelong retention as a standard requirement. Thus, it is imperative for healthcare providers to educate patients about the possibility of relapse after the removal of retention equipment, and the physiological adaptations that occur over time. The lack of concrete evidence and set guidelines regarding the ideal duration of retention and recall intervals highlights the importance of orthodontists avoiding a paternalistic approach.

On the contrary, involving patients actively in discussion about whether to end or extend the retention phase is essential. Maintaining good oral health over an extended long period will not solely rely on patients' effort. Although only a quarter of the population recognizes general dentists as having a role in caring for retention devices, extended retention should involve active participation or at least, effective communication with dental practitioners. The reason behind the Swiss population's preference for lifelong retention is still subject to interpretation. The findings of this study indicate that a vast majority of respondents (94.5%) prioritize achieving consistent results, with many willing to take financial responsibility and personal accountability to guarantee stability. A high proportion of the participants (87.3%) expressed their willingness to engage for a minimum one-year retention term, with a considerable portion anticipating a longer duration.

A significant proportion of survey respondents expressed agreement with recall intervals from three to 12 months, with 73% indicating their readiness to assume the financial burden associated with these subsequent appointments. It is important to note that only half of participants explicitly stated their belief that the patient holds the responsibility for maintaining stability after orthodontic treatment. Nonetheless, the overall results indicate that the majority of respondents understand the need for a certain degree of commitment in this regard.

While orthodontists play a crucial role in providing a stable outcome, it is essential for potential patients or their guardians to recognize the importance of maintenance and compliance to achieve this goal. Based on these observations, it can be concluded that orthodontists are likely to encounter compliant individuals in the post-orthodontic patient population who are willing to actively engage in the retention phase, thus positively affecting its overall effectiveness. The study also revealed a positive correlation between female participants and individuals with higher education levels, showing a greater inclination to cover the cost of recall visits. Although there are limitations in interpreting these findings, they are consistent with previous studies that indicate a positive correlation between higher education levels and a greater willingness to invest in healthcare services. Moreover, these findings align with prior research suggesting that women exhibit more generosity and pro-social behaviors in financial matters compared to men. This study assesses the existing knowledge and expectations of decision-makers before receiving orthodontic information. Conducting a comparative analysis between this study and previous research focusing on patients already undergoing treatment would be a valuable endeavor. This analysis aims to ascertain whether patients or their decision-makers modify their expectations following additional information [34].

Valuable Outcomes From Routine Trials

A qualitative study was undertaken by Tsichlaki A et al. (2020) to explore why individuals sought orthodontic treatment, along with their viewpoints and expectations regarding orthodontic treatment [35]. The study gathered information from a group of teenagers across five separate research sites situated in different regions of England. These facilities provided orthodontic treatment options covered by both the National Health Service and private funding. Participants were selected based on their specific phase within the orthodontic treatment process. Focus groups and interviews were scheduled in advance to accommodate all participants and were held in non-clinical settings by the principal investigator (A.T.), who had received formal training in qualitative research methods. Interviews followed a semi-structured approach based on the main research questions and the scoping review.

The participants were divided into two age groups: 10-13 years and 14-16 years. Additionally, they were categorized according to the stage of orthodontic intervention they were undergoing, including pretreatment, mid-treatment, or post-treatment stages. Initially, the aim was to have a participant pool of approximately 25 to 35 individuals based on previous research. However, the sample size was flexible to accommodate new insights until data saturation was reached.

The research team tested the interview guide and made necessary adjustments. Data was collected using a digital sound recorder to record all interviews and discussions, while field notes were taken immediately after each session. The interview data was transcribed verbatim and then analyzed by two researchers, known as A.T. and F.B.C.-S. The analysis utilized the framework technique. The qualitative data was organized into themes, which were then compared with findings from a previous scoping review. The results were categorized into specific groups and later reviewed with the Study Advisory Group (SAG), comprising 54 orthodontists and 52 dental public health specialists. The SAG members thoroughly assessed each outcome and domain. Duplicate outcomes were merged where appropriate. The results from the scoping review had been previously published.

In summary, 164 trials were reviewed, focusing on outcomes such as pain, periodontal health, and changes in tooth angulation and/or inclination. The studies showed a lack of consistency in the instruments used to measure these outcomes. The study included 35 individuals, with an average age of 14.37 ± 1.23 years, selected from five healthcare facilities in England (2 primary care and 3 secondary care sites). The participants were about to start orthodontic treatment (20%), were currently undergoing treatment (49%), or had finished treatment (31%).

The process of collecting data encompassed the implementation of 7 focus groups and 16 qualitative interviews at specific locations. Participants consistently expressed concerns about several aspects of their oral health-related quality of life (OHRQoL), indicating a significant desire to improve these aspects through appropriate treatment. The research identified five key factors related to treatment outcomes: dental aesthetics, oral function, social interactions, psychological and emotional well-being, and perceived lasting benefits. Additionally, various secondary topics were also noted. The SAG participated in discussions aimed at converting all identified issues into measurable outcomes. The results from the two rounds of analysis were compared and refined to produce a final list of 34 outcomes, which were then organized into 10 distinct domains. In order to make the information more accessible to younger patients, simple explanations were provided below each statement. The e-Delphi survey was conducted online, involving 274 individuals who completed the second round successfully. The overall response rate for the study was 58%. The study included 274 participants, comprising 50 individuals receiving orthodontic treatment, 28 general dentists, and 196 orthodontic clinicians representing 64 countries. The study found that a significant number of participants came from the United Kingdom (22%) and the United States (20%), while other countries each contributed no more than 5% of the total participant responses. Researchers analyzed and discussed forty-five open-ended responses submitted by participants in the initial round. However, these responses did not yield any new or additional results. Therefore, no additional outcomes were added in the subsequent round. By the end of the round, 15 outcomes were recognized to have achieved consensus, but five outcomes were found to lack consensus. The 14 remaining outcomes were categorized as 'consensus out' based on the established scoring criteria. The findings indicated minor differences in responses between participants who completed the task successfully and those who did not. These differences were minimal, with values not exceeding 2 points on the response scale. Regarding the impact of social interactions, a significant gap of 1.8 was observed on the response scale between individuals who completed the dentist group program and those who did not.

While the orthodontic group had unanimously agreed on including this result, the patient group did not reach a consensus. Therefore, any scoring modifications among dentists would not have affected the categorization of this result as 'lack of consensus.' The observed scores on the response scale showed a marginal difference of only one point between participants who finished the study and those who did not, indicating no significant differences in opinions between the two groups. This suggests that attrition bias did not significantly influence the results. Fourteen participants took part in the consensus meeting, with eight individuals (consisting of four patients and four healthcare professionals) eligible to vote. Participants successfully concluded both iterations of the Delphi poll. Each outcome was systematically presented and discussed using the consensus matrix grouping. Participants reviewed six outcomes on the designated day, with the impact on emotions/feelings consistently rated as 'critical' (7-9) by all stakeholders, indicating a consensus on this specific result. The remaining five results underwent a re-voting process and were ultimately categorized as 'out.' However, the consensus conference participants collectively determined that certain outcomes, such as root resorption and skeletal connection, which patients may not be familiar with, would require further discussions with the Stakeholder Advisory Group (SAG) before a final decision is made on their inclusion in the Core Outcome Set (COS). Subsequent discussions with the SAG led to the evaluation of the 16 outcomes [35].

The described variables were categorized into four separate domains using the taxonomy proposed by Dodd S et al. (2023) [40] for creators of Clinical Outcome Assessments (COS). The COS comprises seven discrete outcomes; assessing self-perceived esthetics, evaluating alignment and occlusion, analyzing skeletal relationships, examining stability, identifying fractures, determining adverse effects on teeth or their supporting structures, and evaluating patient adherence. These outcomes align with four key areas: perceived health status, clinical outcomes, adverse events, and care delivery. In the domain of adverse events, two potential consequences were examined: fractures and detrimental impacts on dental structures, particularly teeth and the systems that support them. The rationale behind incorporating a broader range of specific outcomes, such as demineralization, periodontal effects, and root resorption, was to avoid limiting the scope and future potential applications of the COS. It is crucial to distinguish between fractures caused by operators and those linked to patients. The latter specifically relates to patient adherence, which is evaluated within a separate outcome domain. Fractures in this domain are believed to be caused by operators and are significant for research on different bonding methods, cement strength, as seen in the Cochrane review on adhesives used for fixed orthodontic brackets [41]. Furthermore, fractures may also include potential failures in appliances or components, such as temporary anchorage devices. The care delivery domain focuses solely on one result derived from a set of more precise outcomes related to patient adherence. The significance of patient adherence is underscored in the overall assessment of the treatment procedure. Adherence assessment can be performed using objective and subjective approaches, employing metrics from clinicians or patient-reported outcome measures.

In a recent study on orthodontic adherence, the assessment of usage and adjuncts of removable orthodontic appliances indicated that subjective evaluations led to an overestimation of the duration of device or adjunct wear [35]. Overall adherence rates were also found to be suboptimal, highlighting the need for more interventional studies to improve understanding and factors related to adherence in this specific domain. The ultimate focus of the core outcome set was a combined measure that looked at how one's personal perception of aesthetics influences their health status. The outcome arose from merging 'self-perceived aesthetics' and 'impact on emotional well-being,' indicating how orthodontic treatments can affect OHRQOL. In interviews and focus groups, young patients consistently stressed the importance of outcomes related to how they view their perception of their appearance and how it affects their social and emotional well-being. However, patient stakeholders did not express support for including 'impact on social well-being' in the final decision-making process. This result could be due to variations in the age range of participants involved in the Delphi consensus and qualitative interviews [35].

Orthodontists engaged in the Delphi process consistently assigned significant ratings to these outcomes. There is a growing acknowledgement among practitioners and researchers about how malocclusion and orthodontic intervention can influence OHRQOL. The recognition of this phenomenon is driven by the escalating focus on evaluating treatment outcomes from the patient's perspective and the need to validate treatment effectiveness through measurable advantages. Therefore, there is an increased recognition of the importance of including assessments of OHRQOL in future research activities. It is imperative to conduct further rigorous clinical trials that employ appropriately selected patient-reported outcome measures to examine how self-perceived esthetics impact patients' overall well-being [35].

Furthermore, Angst C et al. (2021) undertook a study involving a group of fifty individuals aged between 11.9 and 34.1 years [36]. The median age of the cohort was 14.3 years, with an interquartile range of 13.4 to 15.2 years. Within this cohort, 60% were females. Findings revealed that half of the patients underwent tooth extractions, with 8 patients having two upper teeth extracted and 17 patients having four premolars removed. The remaining 50% did not receive extractions and were followed up at debond Time 1 (T1) for a median duration of 8.0 years, with an interquartile range of 7.2 to 9.2 years, and an overall range from 6.4 to 13.5 years. This study included patients who attended their check-ups before either too or after the specified seven or 10-year intervals. There were no statistically significant differences observed in relation to gender or duration of follow-up between patients who underwent extraction and those who did not. However, the research findings showed a significant difference in the age distribution of individuals who underwent extraction at T1 compared to those who did not. The average ages of patients who underwent extraction and those who did not were 15.2 years and 13.4 years, respectively (P = 0.001). The mean ABO score at T1 was 37.6 ± 9.3 points. Individuals who received tooth extraction showed significantly superior finishing outcomes compared to those who did not have extractions, as indicated by the ABO scores of 34.2 and 40.9 points respectively (P = 0.009). In contrast, no statistically significant correlation was found between patients' age at T1, their gender, and the ABO score at T1 (P = 0.19 and P = 0.82, respectively). Following a median duration of 8.0 years, the ABO score at in-retention (T2) had a mean value of 30.2 ± 10.3 points. It is noteworthy to emphasize that no statistically significant changes were seen between individuals who underwent extraction and those who did not (P = 0.29). The results suggest a statistically significant decrease in ABO scores from T1 to T2, with an average reduction of 7.4 points. The results suggest an overall decline in a specific measure, specifically alignment/rotations on average. However, the study consistently observed two specific criteria, namely occlusal connections and interproximal contacts, which exhibited persistent patterns. Conversely, significant enhancements were observed in four distinct variables: marginal ridges, buccolingual inclination, overjet, and occlusal contacts. There was no statistically significant correlation found between patients' age at T1, their gender, and the length between T1 and T2 with the overall change in ABO levels between T1 and T2. Nevertheless, a significant correlation was found between the initial ABO score at T1 and the subsequent change in ABO score from T1 to T2. Patients with higher ABO scores at T1 demonstrated a more significant improvement in ABO during the ensuing observation period (P = 0.001). Upon undertaking a comprehensive analysis of individual patients, it was discovered that the collective ABO score exhibited a consistent improvement over an 8-year observation period in the majority of cases, encompassing around 82% of the total instances. In contrast, a small proportion of patients (16%) experienced a reduction in their ABO score, while a single patient (2%) showed no change in their score. Regarding each specific ABO criterion, most cases exhibited an improvement in marginal ridges, buccolingual inclination, overjet, and occlusal contacts, or a decline in alignment/rotation. Concerning the final criterion of occlusal connections, it was observed that around 40% of subjects experienced enhancements or declines, and the remaining 20% showed stability. Several influential factors significantly contributed to the progress following T1, particularly with regard to two key criteria. These factors encompassed the integration of extractions into the treatment plan and adherence to ABO criteria for cases at T1. The analysis revealed that cases involving extractions exhibited a significantly higher likelihood of improvement (28% as opposed to 0%) and a notably lower likelihood of worsening (52% compared to 96%) regarding marginal ridges compared to non-extraction cases. Furthermore, it was observed that cases classified as 'ABO pass' at T1 had a higher likelihood of improvement in occlusal relationships compared to those who did not meet the 'ABO pass' criteria (64% versus 28%). The only instances where a decline was observed following debonding were in the areas of alignment/rotation and occlusal connections, with 58% and 38% of participants experiencing deterioration, respectively. The phenomenon of alignment/rotations can be readily explained by the common occurrences of mesial migration and tertiary crowding during the initial phases of maturation. One significant finding of this research was the impact of improved finishing quality during the debonding process on the long-term outlook of the dentition. The study revealed that this enhancement had a discernible impact on the improvement of diverse ABO measures. The preliminary findings revealed a statistically significant positive association between ABO scores during debonding and the likelihood of worsening in the alignment/rotations criterion (P<0.001). This suggests that proper teeth alignment during the dental procedure may inhibit the anterior movement of teeth, hence contributing to dental overcrowding.

Furthermore, an analysis of the participants who met the criteria set by the ABO at T1 revealed that they were more likely to experience improvements, with 64% showing improvements in retention, 21% maintaining stability, and 14% experiencing deterioration. The statistical analysis yielded a significant difference in ABO failures at T1 (P= 0.02). In contrast, the present study's results reveal that 53% of patients experienced a decrease in retention, 28% showed improvement, and 19% maintained stability. It is possible that established occlusal relationships with antagonists could lead to increased stability or improvement post-debonding [36].

Some scholarly papers have suggested that adhering to ABO norms in post-orthodontic dental occlusion can result in a more consistent activation of the anterior temporalis muscle during functional activities, and improved perceived chewing function.

The application of a 30-point threshold for the ABO score as an indicator of treatment efficacy presented particular challenges due to the subjective nature of classifying cases as "successful" or "unsuccessful". Upon reevaluation, it was found that 50% of cases that did not meet the ABO standards following debonding eventually fulfilled these criteria during retention. Furthermore, around one-third of cases initially considered successful based on the ABO criteria experienced a decline in retention, subsequently classified as failures.

In a cohort of eight patients showing positive T2-T1 changes in ABO score, indicating a decrease in occlusion, four fulfilled the ABO criteria upon removal of orthodontic brackets, while four did not. This raises questions about the sufficiency of this criterion in terms of clinical or biological justifications. The findings of this study indicate that a specific cohort of patients underwent premolar extractions, experienced a significant impact on the occlusal outcome. Patients undergoing extractions during the debonding process demonstrated considerably better outcomes compared to those who did not have extractions (P = 0.009), indicating a potentially impactful relationship between extractions, stability, and dental alignment post-orthodontic treatment. This finding contradicts prior research showing similar PAR scores and anterior dental crowding between patients who underwent extractions and those who did not. It is crucial to note that the previous study had a limited follow-up duration of 2.4 years and used the PAR index for occlusal evaluation, which may not capture the intricate nuances of occlusion as effectively as the ABO tool [36].

Quality-of-Life (QoL) Measurements

The results of Grewal H et al. (2019) demonstrated a satisfactory to strong level of internal consistency among the items in each domain [37]. The statistical analysis employed the Wilcoxon signed-rank test. Findings showed that many domain scores exhibited an over-dispersed pattern, leading to the use of a negative binomial regression model. The study considered covariates such as time (before and after orthodontic treatment), gender, and malocclusion class. It also investigated the correlation between time and gender, and socioeconomic status, to evaluate any potential changes in domain scores over time. Results indicated significant differences in domain score distributions before and after orthodontic treatment across all domains, with overall improvement during treatment. Significant disparities were observed in domain scores pre- and post-treatment.

The decision to incorporate the negative binomial link function in the generalized estimating equation (GEE) was driven by the observation that, in several contexts where zero values are present, the variance typically tends to exceed the mean. Lower scores were considered preferable in all other categories except for dental self-confidence. Conversely, higher scores were viewed as more positive in terms of dental confidence. The findings of the negative binomial regression analysis revealed a statistically significant enhancement in all domains during orthodontic treatment. In the adjusted analysis, there was no significant interaction between gender and time across all categories. However, a significant relationship was observed between time and malocclusion class. Gender and socioeconomic position did not appear to have a significant impact on the changes in scores across all domains throughout the specified timeframe. The degree of malocclusion showed variations exclusively in relation to functional factors. The selection of young adults was based on their high level of awareness and practical approach toward dentofacial aesthetics. Furthermore, the researchers considered their ability to effectively assess the psychosocial effects of dental aesthetics.

The findings of the study indicate a discernible change in the perception of dental aesthetics as individuals age, leading to an overall improvement in this perspective. Based on the existing findings, there is evidence suggesting a decline in the perceived significance of orthodontic treatment demands as a person progresses in age. Therefore, a comprehensive evaluation was carried out to determine the extent of malocclusion (Angle's class I, II, III) in the sample, with a particular emphasis on functional limitations.

Functional improvement was shown in all types of malocclusions after orthodontic treatment. The research revealed that individuals with Angle's Class III malocclusion experienced a higher level of psychosocial impairment prior to receiving therapy compared to individuals with other forms of malocclusion. Additionally, it was noted that the class III group showed a relatively lesser degree of improvement in functionality compared to class I and class II malocclusion groups. However, it is crucial to recognize that significant enhancements were identified across all three groups. The psychological benefits of orthodontic treatment were positive across various areas, except in the marital problems domain, where improvement, particularly among males, was not significant. This suggests that the psychological effects of malocclusion on Indian males regarding marital concerns are relatively restricted, compared to those experienced by females [37].

A study undertaken by Paes da Silva S et al. (2020) provided evidence to support this conclusion. The study examined a dataset of 666 complete cases to evaluate how various model parameters, such as age group, gender, reason for treatment, type of appliance, treatment duration, self-esteem, insurance, and migration status, influenced the OHRQoL of patients [38].

The focus of the study was on the total Oral Health Impact Profile (OHIP) score. The study identified several variables that significantly affected the individual OHIP scores of patients. Being categorized as an adult, defined as individuals aged 18 years or older, was significantly associated with the OHIP score (P<0.038). Gender, particularly being female, showed a significant relationship with the OHIP score (P=0.008). The presence of fixed appliances, such as braces or other orthodontic devices, demonstrated a highly significant association with the OHIP score (P<0.001). Reasons underlying the pursuit of treatments, including aesthetic concerns, pain, and other factors, were found to have a statistically significant impact on treatment outcomes. A decline in self-esteem was found to be significantly associated with the outcome variable (P<0.001).

The characteristics were positively correlated with the OHIP score, leading to a negative effect on the OHRQoL of patients. Variables such as treatment duration, insurance coverage, and immigration status did not show a significant association with the OHIP score. The study provided OHIP values for the entire patient group (N=666), and separately for children (N=74), adolescents (N=530), and adults (N=227). The study aimed to explore the correlation between model parameters and their impact on the QoL, as assessed by the OHIP-G14, across different age groups, including children, adolescents, and adults. In Germany, governmental insurance coverage is determined by factors such as age and malocclusion severity. Governmental insurance is available for those under the age of 18. Adults in Age group 3 are only eligible for this coverage in specific situations, such as requiring orthognathic surgery and orthodontic treatment together. These conditions serve as exclusion factors, leading to separate multiple linear regression analysis for each age group. No significant influences on the OHIP score were found in all age groups or specific age groups when considering 'aesthetics', 'function', 'pain', or 'others' elements separately. However, notable enhancements in the OHIP score were observed in combinations with at least three elements within the 'all age' group, encompassing both adolescents and adults. Fixed appliances significantly increased the OHIP score across all age groups (P=0.006). In the adult model, the ratio of 'removable' appliances to 'permanent' appliances was 1:2.8, while in the adolescent model, it was 1:2.3. To effectively tackle this variability, a comprehensive reevaluation of the teenage model was undertaken. The present study employed a randomized selection process to analyze 227 individuals within a specific age group. The 1:2.8 ratio of adults was employed to ensure the selection of a representative sample.

Results from linear regression models were compared, revealing significant differences, particularly among female patients. While most models showed no significant relationship with the OHIP score, some predictors were significant, especially among adolescents. In the majority (70%) of the models examined, the predictor under investigation did not demonstrate a statistically significant relationship with the observed increase in patients' OHIP ratings. Nevertheless, the predictor under consideration had a statistical significance level that was somewhat below the customary criterion (P=0.049) among the group of adolescent patients [38].

The current study, including 898 individuals, both young adolescents and adults, aimed to assess the influence of orthodontic intervention on OHRQoL. Multiple variables were examined to evaluate these effects thoroughly.

The study revealed that the average OHIP score among adults was 12.6, while adolescents averaged 8.9. The findings align with prior research involving diverse sample populations. The OHIP-G14 questionnaire highlighted physical pain and psychological discomfort as significant factors across children, adolescents, and adults, negatively affecting their OHRQoL. The study revealed that orthodontic intervention for severe malocclusions led to improved cosmetic outcomes and enhanced OHRQoL, particularly in reducing psychological distress and functional impairment. Three important characteristics were identified concerning how patients perceive their OHRQoL.

Many individuals express concerns about the aesthetics of their teeth, affecting their social interactions, oral health, and daily activities. These concerns were validated by the results of the survey. The OHIP-G14 tool is widely employed to evaluate how various factors affect individuals' QoL. Multiple linear regression models revealed significant influences of different variables on OHRQoL across all age groups (children, adolescents, and adults), rejecting the null hypothesis of equal variable impacts. Adult patients in the study group experienced a significant increase in OHIP scores. Researchers suggested orthodontic treatment could potentially affect adults' QoL negatively, promoting further investigation. Females across all age groups tended to report lower QoL or exhibit a tendency towards self-criticism. Age groups were primarily categorized based on insurance coverage and the prevalence of references to the OHIP-G14 in academic discourse.

In Germany, individuals who are 17 years old or younger receive subsidized orthodontic treatment through a government-funded insurance system. Adults are responsible for covering their own treatment costs or seeking coverage through private insurance. The OHIP-G14 is commonly cited in scholarly literature for age group categorizations. Upon the initial investigation, it was found that younger individuals might struggle with responding to the questionnaire. Therefore, parents and caregivers may need to offer assistance if needed. However, accurately determining the level of parental support for children aged 11 years or younger is challenging due to the questionnaire's self-administered and anonymous nature. This challenge may not directly relate to OHIP-G14 inquiries, but instead to inquiries about insurance status.

The validation process for the German adaptation of the OHIP-14 encompassed a diverse patient cohort ranging from 16 to 79 years old. Extensive literature analysis was undertaken to investigate the application of the OHIP in orthodontics. Several academic articles have utilized the OHIP to evaluate patient groups in similar age ranges. Recent research has included the OHIP-14 instrument in investigations involving adolescents. Scholars generally agree on the suitability of utilizing the OHIP-14 questionnaire for adolescents aged 12 to 17 years and adult populations.

It is important to acknowledge that the questionnaire has also been employed in patients who were under the age of five. This study examined a representative sample to determine key elements affecting patients' OHRQoL. Aesthetic concerns, functional challenges, and pain, collectively termed the 'reason for treatment,' significantly affect OHRQoL. A multiple linear regression model demonstrated that aesthetics, function, and pain significantly influence the OHRQoL of adolescent patients. In contrast, functional impairments and pain are notable factors for adult patients. These concerns serve as essential indicators for orthodontic intervention across all age groups, stressing the importance of seeking orthodontic treatment from experts. Therefore, it is unsurprising to witness higher scores in these particular categories of the OHIP, as they are strongly linked to the essential nature of orthodontic treatment. Orthodontic treatment aims to improve both the aesthetic and functional aspects of an individual's dentofacial characteristics, as well as address pain management and psychological well-being. Patients in this specific cohort consider not only visual appeal but also functionality and comfort when considering orthodontic intervention. If patients' expectations, such as pain relief during orthodontic treatment, are not met, they may report higher OHIP ratings, indicating a decrease in OHRQoL [38].

## Conclusions

Substantial progress has been made in orthodontic retention over the last five decades. Understanding of potential post-orthodontic relapse and the importance of post-orthodontic retention varies and appears to be influenced by sociocultural factors. There is a prevalent consensus among individuals in Switzerland who are considering orthodontic treatment or involved in the decision-making process. This consensus encompasses various aspects, including the distribution of accountability, the perceived importance of follow-up visits, the assumption of financial costs, and the preferences for retainer devices.

Orthodontists commonly schedule retainer check-ups for 1-2 years after the completion of active orthodontic treatment. While the dominant perspective supports the continuation of retention practices, some orthodontists express concerns regarding the involvement of general dentists in assessing retention for patients with retainers. The production of retainers is mostly conducted in orthodontists' office laboratories, with variances in retainer characteristics observed among different practitioners. The presence of various retention approaches among orthodontists underscores potential challenges in implementing a standardized retention protocol for all patients. Customizing key outcome measures is crucial to correspond with the distinct characteristics of each orthodontic research study, fitting the diverse range of orthodontic treatments and trial designs. Research has demonstrated that orthodontic intervention leads to positive psychological outcomes across various psychological evaluation domains, thereby improving overall OHRQoL. Orthodontic treatment has resulted in significant improvements in functional and aesthetic characteristics for various malocclusion categories. Both genders exhibit similar impacts on overall well-being before and after orthodontic intervention.

A consensus has been reached on the essential themes and outcomes that should be included in clinical trials related to orthodontic retention for non-cleft and nonsurgical cases.
